# Advances in deciphering the genetic basis of insect cuticular hydrocarbon biosynthesis and variation

**DOI:** 10.1038/s41437-020-00380-y

**Published:** 2020-11-02

**Authors:** Henrietta Holze, Lukas Schrader, Jan Buellesbach

**Affiliations:** 1 Molecular Evolution and Sociobiology Group, Institute for Evolution and Biodiversity, University of Münster, Hüfferstr. 1, DE-48149 Münster, Germany; 2 Department of Environmental Science, Policy, and Management, University of California—Berkeley, 130 Mulford Hall #3114, Berkeley, CA 94720-3114 USA

**Keywords:** Chemical genetics, Genetics, Evolutionary genetics, Evolutionary ecology

## Abstract

Cuticular hydrocarbons (CHCs) have two fundamental functions in insects. They protect terrestrial insects against desiccation and serve as signaling molecules in a wide variety of chemical communication systems. It has been hypothesized that these pivotal dual traits for adaptation to both desiccation and signaling have contributed to the considerable evolutionary success of insects. CHCs have been extensively studied concerning their variation, behavioral impact, physiological properties, and chemical compositions. However, our understanding of the genetic underpinnings of CHC biosynthesis has remained limited and mostly biased towards one particular model organism (*Drosophila*). This rather narrow focus has hampered the establishment of a comprehensive view of CHC genetics across wider phylogenetic boundaries. This review attempts to integrate new insights and recent knowledge gained in the genetics of CHC biosynthesis, which is just beginning to incorporate work on more insect taxa beyond *Drosophila*. It is intended to provide a stepping stone towards a wider and more general understanding of the genetic mechanisms that gave rise to the astonishing diversity of CHC compounds across different insect taxa. Further research in this field is encouraged to aim at better discriminating conserved versus taxon-specific genetic elements underlying CHC variation. This will be instrumental in greatly expanding our knowledge of the origins and variation of genes governing the biosynthesis of these crucial phenotypic traits that have greatly impacted insect behavior, physiology, and evolution.

## General characteristics of cuticular hydrocarbons

One main contributing factor to the remarkable evolutionary success of insects, eventually becoming the most diverse and species-rich class in the animal kingdom (Grimaldi and Engel 2005), has been attributed to their exoskeleton and their cuticle (Nation 2008; Kather and Martin 2015, Fig. 1). Within this structure, the waxy lipid layer on the cuticle’s outer segment (i.e., “epicuticle”) is the part that directly interacts with the environment and has thus been exceptionally well investigated (Lockey 1988; Nelson and Blomquist 1995; Nation 2008). It is composed of a complex mixture of alcohols, esters, aldehydes, ketones, long-chain fatty acids (LCFAs), and long-chain hydrocarbons (Wigglesworth 1933; Blomquist et al. 1987). The latter compound class constitutes the dominant fraction, termed cuticular hydrocarbons (CHCs) (Lockey 1980; Howard and Blomquist 1982).

**Fig. 1:**
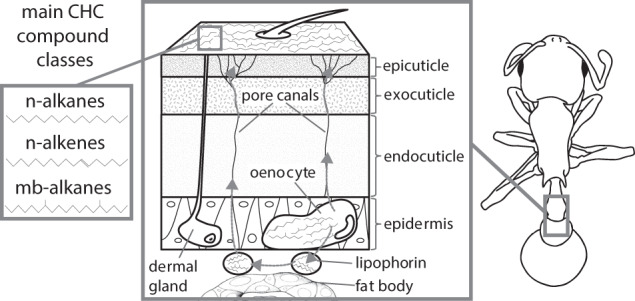
Right: Schematic insect, middle: Cross-section through its integument highlighting CHC transport pathways and their deposition on the epicuticle, left: examples of the three most commonly occurring CHC compound classes *n*-alkanes, *n*-alkenes, and methyl-branched alkanes. CHC biosynthesis occurs in specialized secretory cells, the oenocytes, which are mainly embedded in clusters in the epidermis or dispersed within the fat body depending on insect species and developmental stage. After their biosynthesis, CHCs are shuttled through the hemolymph by the high-density lipoprotein lipophorin, with subsequent transport to the epicuticular surface via specialized pore canals penetrating the cuticular layers. Gray arrows indicate CHC transport pathways, drawings by Lukas Schrader.

CHCs display a wide range of distinct compound blends varying considerably across different insect taxa (Lockey 1988; Blomquist and Bagnères 2010; Sprenger and Menzel 2020). CHC profiles have been found to differ by number of compounds, their respective proportions, chain lengths, and chemical compositions (Lockey 1988; Howard and Blomquist 2005). In most insects, linear straight-chain hydrocarbons (i.e., *n*-alkanes) are one of the major CHC compound classes (Blomquist et al. 1987), with chain lengths typically greater than 20 carbon atoms (Gibbs and Pomonis 1995; Blomquist and Bagnères 2010). Alkanes with methyl groups (i.e., methyl-branched alkanes) also constitute abundant CHC components in most studied insect species (Blomquist and Bagnères 2010). Finally, unsaturated hydrocarbons with one or two double bonds, (i.e., *n*-alkenes and alkadienes, respectively) have been found to occur quite frequently in a number of insect taxa as well (Blomquist et al. 1987; Blomquist and Bagnères 2010). Representative examples of the major CHC compound classes are given in Fig. 1. Other compound classes, e.g., unsaturated hydrocarbons with three or four double bonds (i.e., trienes and tetraenes, respectively) or simultaneously possessing double bonds and methyl groups (i.e., methyl-branched alkenes), have been found to occur only sporadically in comparably few insect CHC profiles (Blomquist et al. 1987; Kather and Martin 2015).

CHCs primarily serve as protection barrier against desiccation and could be one of the factors that facilitated the colonization of dry land by insects (Blomquist and Bagnères 2010). Furthermore, CHCs are principal signals in chemical communication (Carlson et al. 1971; Blomquist and Bagnères 2010). As versatile semiochemicals, they can encode and transmit a wide variety of information including but not limited to reproductive status (e.g., Smith and Liebig 2017), species affiliation (e.g., Shahandeh et al. 2018), sex (e.g., Luo et al. 2019), age (e.g., Heuskin et al. 2014), and social rank (e.g., Honorio et al. 2019). Moreover, in eusocial insects, CHCs are fundamental as the major nestmate and caste recognition cues (Leonhardt et al. 2016). CHCs have also been shown to play pivotal roles in sexual communication. They constitute the main cues to attract conspecific mates (e.g., Heggeseth et al. 2020), to trigger courtship and copulation behavior (e.g., Buellesbach et al. 2018a), and to signal receptivity, fertility, and mating status (e.g., Billeter and Wolfner 2018).

## Basics of CHC biosynthesis

Despite considerable diversity of CHC blends across different insect taxa, the fundamental biosynthetic pathway of CHC production appears to be highly conserved and linked to fatty acid biosynthesis (Blomquist and Bagnères 2010, Fig. 2). Insects generally synthesize the majority of the components in their CHC profiles themselves (Nelson and Blomquist 1995; Blomquist and Bagnères 2010), with much smaller quantities of dietary hydrocarbons being directly incorporated into their epicuticle (Blomquist and Jackson 1973). In eusocial insects, individually synthesized CHCs can also be socially transferred to nestmates (Blomquist and Bagnères 2010; Leonhardt et al. 2016). CHCs are mainly produced in oenocytes, specialized secretory cells that are associated with the epidermal layer or peripheral fat body, depending on insect species and developmental stage (Wigglesworth 1933; Schal et al. 1998, see Fig. 1). The general pathway consists of the elongation of fatty acyl-Coenzyme A units to produce very-long-chain fatty acids (VLCFs) that are subsequently converted to hydrocarbons by subducting the carboxyl group (Nelson and Blomquist 1995; Howard and Blomquist 2005; Blomquist and Bagnères 2010, Fig. 2).

**Fig. 2:**
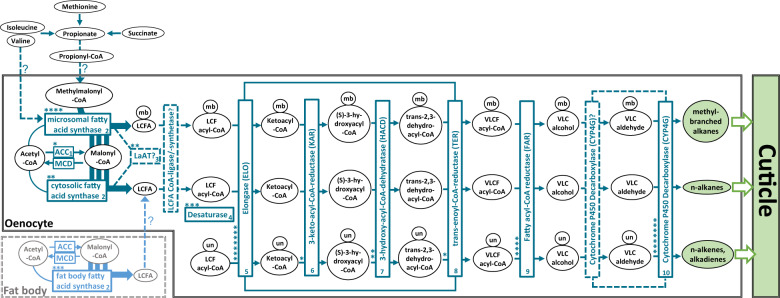
Schematic summary of the current state of knowledge for the CHC biosynthesis pathway in insects. Circles designate chemical compounds, rectangles the corresponding enzymes catalyzing their transitions. Enzymes are numbered from 1 to 10 according to their hypothesized order in the pathway, and asterisks correspond to the respective number of characterized genes whose depicted function has been empirically demonstrated through targeted knockdown studies listed in Table 1. Reactions and interactions in the CHC biosynthesis pathway that are not completely understood are marked with dashed arrows and question marks. Acetyl-CoA as the initial reactant of CHC biosynthesis is mainly provided by the citric acid cycle. Note that the distinction between microsomal and cytosolic fatty acid synthase is hypothetical and has not yet been unambiguously confirmed. Abbreviations: **CoA**: Coenzyme A, **ACC**: acetyl-CoA carboxylase, **MCD**: malonyl-CoA decarboxylase, **LaAT**: lipoamide acyltransferase, **LCF(A)**: long-chain fatty (acid), **VLC(F)**: very-long chain (fatty), **mb**: methyl-branched, **un**: unsaturated. Figure adapted and synthesized from Howard and Blomquist (2005), Blomquist and Bagnères (2010), Chung and Carroll (2015), Ginzel and Blomquist (2016).

At the starting point, acetyl-coenzyme A (acetyl-CoA), which is mainly derived from the citric acid cycle, is converted into malonyl-coenzyme A (malonyl-CoA). This constitutes the main rate-limiting step for the general production of fatty acids and hydrocarbons (Barber et al. 2005). Then, further malonyl-CoA units are successively incorporated onto the acetyl-CoA primer to form LCFAs. Most commonly, myristic acid (14 C), palmitic acid (16 C), and stearic acid (18 C) are produced, with palmitic acid being the predominantly produced LCFA (Blomquist and Bagnères 2010). After that, they are converted to long-chain fatty acyl-CoAs (LCF acyl-CoAs). In the next step, further malonyl-CoA units are incorporated into LCFAs yielding VLCF acyl-CoAs with carbon chain lengths most commonly ranging between 20 and 40 C. However, compounds with longer carbon chains of up to 60 C that usually elude detection with standard analytical methods do occur as well (e.g., Akino 2006; Bien et al. 2019). The synthesis of VLCF acyl-CoAs is followed by the conversion to very-long-chain fatty alcohols (VLC alcohols) and the dehydrogenation to very-long-chain fatty aldehydes (VLC aldehydes). A final decarbonylation step reverts the VLC aldehydes to either *n*-alkanes, methyl-branched (mb-), or unsaturated CHCs (Fig. 2).

For the synthesis of mb-CHCs with internal methyl groups, methylmalonyl-CoA units are incorporated at specific-chain locations instead of malonyl-CoA units (Nelson and Blomquist 1995; Blomquist and Bagnères 2010). In insects with high levels of vitamin B_12_ such as termites, succinate has been shown to be converted to propionate and then through propionyl-CoA as an intermediate to methylmalonyl-CoA (Blomquist et al. 1980; Chu and Blomquist 1980). In insects with low to no levels of vitamin B_12_, propionate and ultimately methylmalonyl-CoA subunits are derived from the externally provided amino acids valine, methionine, and isoleucine (Dillwith et al. 1982; Chase et al. 1990, see Fig. 2). Propionate has also been shown to be synthesized and provided for further synthesis to mb-CHCs by gut microbes (Guo et al. 1991). Externally branched (2-methyl) CHCs represent an exception, as their biosynthesis directly relies on the carbon skeletons of valine (leads to even-numbered mb-CHCs) and isoleucin (leads to odd-numbered mb-CHCs) as precursors (Ginzel and Blomquist 2016). The transport mechanism of these precursor amino acids as well as propionate (or propionyl-CoA) into the oenocytes has not been fully elucidated so far. The introduction of double bonds into the fatty acyl-CoA chain between elongation steps, on the other hand, leads to unsaturated CHCs, most commonly *n*-alkenes and alkadienes (Cook 1985, Fig. 2).

After their biosynthesis, the different CHC compound classes are transported by the high-density lipoprotein lipophorin through the hemolymph with subsequent exposure on the cuticular surface through specialized pore canals (Haruhito and Haruo 1982; Schal et al. 1998, 2001, see Fig. 1). As CHCs remain on shed exuvia after eclosion or molting, the full bouquet of hydrocarbons is replenished after each molt. For this, CHCs are first stored internally before molting and subsequently transferred to the cuticular surface (Howard and Blomquist 2005). It has been postulated that CHC biosynthesis is regulated by an interplay of ecdysteroid hormones and juvenile hormone (Wicker and Jallon 1995; Bilen et al. 2013). Indeed, in one of the earliest genetic studies on CHC variation, Wicker and Jallon (1995) found that the temperature-sensitive ecdysteroid deficiency *Drosophila melanogaster* mutant *ecd-1* expresses altered CHC profiles (Table S1).

## First studies on CHC genetics

CHC-based chemical communication and CHC profile variation have been exceptionally well-studied in the fruit fly genus *Drosophila* for decades (e.g., Jallon 1984; Coyne and Oyama 1995; Ferveur 2005). Several intriguing instances of CHC signaling involved in assortative mating, sexual communication, and prezygotic reproductive isolation have been documented in *Drosophila* (e.g., Coyne et al. 1994; Savarit et al. 1999; Arienti et al. 2010). Therefore, the first investigations of CHC genetics were mostly concerned with unraveling the heritable genetic factors governing sex- and species-specific CHC variation between different *Drosophila* strains and species (e.g., Ferveur 1991; Coyne and Oyama 1995; Ferveur and Jallon 1996).

For instance, in certain *Drosophila* species including *D. melanogaster*, CHC profiles show a clear sexual dimorphism, with male profiles being dominated by *n*-alkenes, mostly 7-tricosene and 7-pentacosene, and female profiles producing much larger quantities of alkadienes, most prominently the female sex-pheromones 7,11- and 5,9-heptacosadiene (Jallon and David 1987; Dallerac et al. 2000; Marcillac and Ferveur 2004). It has been shown that this sex-specific difference is partially controlled by a genetic factor located on chromosome 3, with its specific identity and function remaining elusive (Coyne et al. 1994). Conversely, control of the *n*-alkene ratio has been found to be much more complex. The overall production of 7-tricosene and 7-pentacosene has been found to be additively influenced by two genetic factors mapped to chromosome II, *sept* and *smoq* (Ferveur and Jallon 1996). In addition, 7-tricosene levels, in particular, have been found to also be controlled by multiple genetic factors on chromosome III (Scott and Richmond 1988; Ferveur and Jallon 1996), including nerd (Ferveur and Jallon 1993a, Table S1) and fruitless (Cobb and Ferveur 1995). Interestingly, in the closely related sibling species *D. simulans*, a different locus, *kété*, was found to mostly control 7-tricosene levels (Ferveur and Jallon 1993b), whereas *n*-alkene ratios were found to be predominantly controlled by another single locus, *Ngbo* (Ferveur 1991). These findings highlight how convergent adaptations in CHC profiles can evolve via vastly different genetic mechanisms (Ferveur 2005). Most of these genetic factors, however, have not been characterized any further on a molecular level.

Furthermore, manipulations of several *Drosophila* sex-determination genes have been shown to influence CHC profiles. Mutations as well as ectopic expressions of genes such as *Sex-lethal*, *doublesex* and *transformer* were shown to be capable of both masculinizing female CHC profiles (Jallon et al. 1986, 1988; Tompkins and McRobert 1995) and feminizing male CHC profiles (Tompkins and McRobert 1989; Ferveur et al. 1997; Waterbury et al. 1999, see Table S1). This potentially upstream function of sex-determination genes affecting CHC compositions has been specifically confirmed for transformer in later studies, where ectopic expression of transformer in males directly affected expression of otherwise female-specific CHC biosynthesis genes (Chertemps et al. 2006, 2007). Similarly, mutations in *ovo*, a gene generally regulating ovary development, surprisingly led to increased CHC levels in both males and females (Wicker and Jallon 1995, Table S1). But except for transformer, direct relationships between these sex-specifically expressed genes and molecular mechanisms regulating CHC biosynthesis have not been elucidated any further as of yet.

## Genes directly involved in CHC biosynthesis and variation

### Acetyl-CoA carboxylase (ACC)

The first and rate-limiting step in CHC biosynthesis, the conversion of acetyl-CoA to malonyl-CoA, has been found to be catalyzed by the ACC (Barber et al. 2005, see Fig. 2). ACC specifically binds an acetyl-CoA unit as primer and successively decarboxylates malonyl-CoA subunits to mostly produce C14, C16, and C18 LCFAs (Barber et al. 2005; Blomquist and Bagnères 2010). Direct evidence for the fundamental importance of the ACC gene for CHC biosynthesis in *D. melanogaster* was found by Wicker-Thomas et al. (2015). Targeted RNAi knockdown of that gene in oenocytes resulted in complete CHC depletion for both male and female fruit flies (Table 1). It will be interesting to investigate the universality of ACC as catalyst for the first step in CHC biosynthesis in further insect taxa beyond *Drosophila*, with particular focus on other genes potentially capable of complementing this vital function. Intriguingly, in the jewel wasp *Nasonia vitripennis*, a highly expressed malonyl-CoA decarboxylase was found to reverse the metabolic function of ACC by degrading malonyl-CoA to acetyl-CoA (Sacksteder et al. 1999; Lammers et al. 2019, see Fig. 2). However, this has only been studied in the context of fatty acid metabolism, and the direct impact of this gene on CHC biosynthesis remains to be investigated.

**Table 1:**
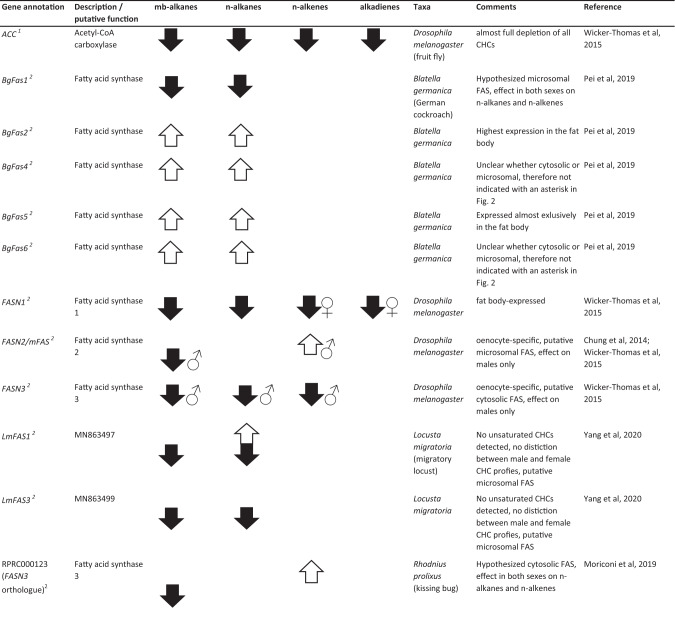

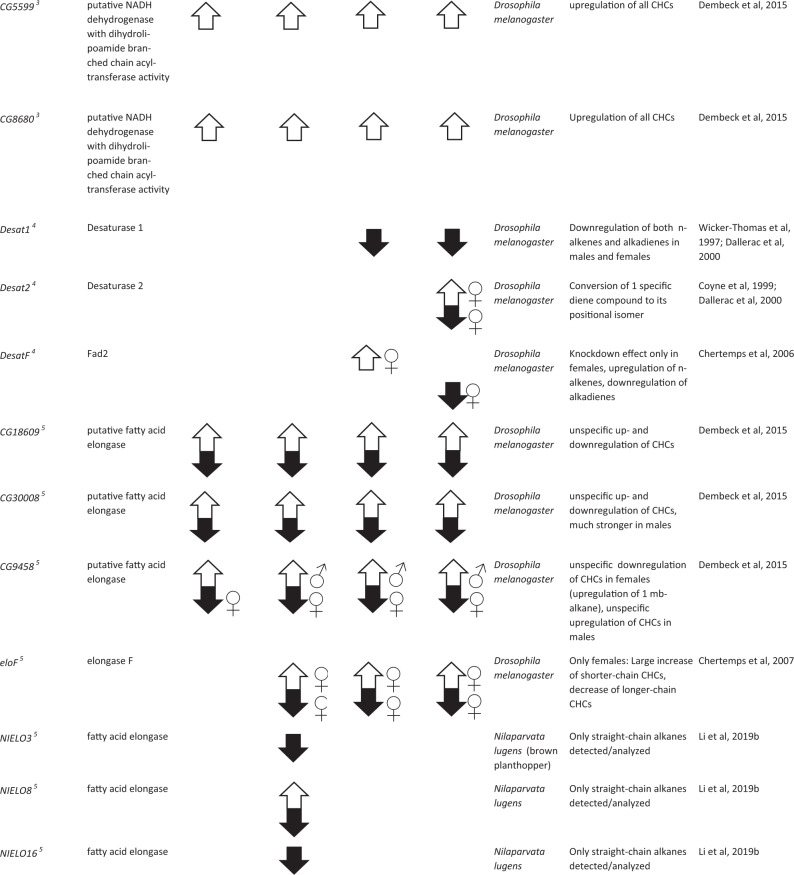

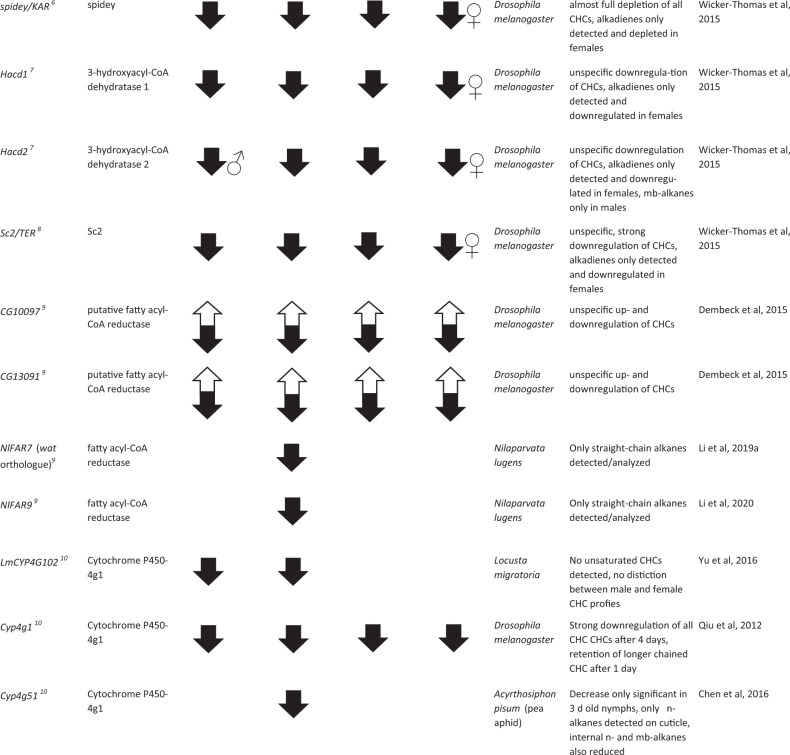
List of 35 genes whose functions could umambiguously be assigned to the CHC biosynthesis pathway through targeted knockdown/mutation studies (compare to Fig. 2), separated according to their effects on the four major CHC compound classes mb-alkanes, *n*-alkanes, *n*-alkenes, and alkadienes. Up (white) and down (black) arrows correspond to either up- or down- regulated CHC production in each of the four compound classes after gene knockdown/mutation, male and female symbols indicate sex-specific effects if not noted otherwise in the comments. Annotations, descriptions, and putative gene functions were retrieved from NCBI where possible, or from the indicated references. Superscript numbers next to the gene names correspond to the hypothesized order in the CHC biosynthesis pathway as represented in Fig. 2. More information on the genes such as estimated evolutionary rates based on orthogroup and phyletic profiles can be found in Table S1 in the supplementary information as well as more genes with an impact on CHC profiles whose function could not be assigned to a particular step in the CHC biosynthesis pathway.

### Fatty acid synthase (FAS)

It has been speculated that insects generally possess two types of FASs, cytosolic and microsomal, although no direct experimental evidence for their exact subcellular localization could be delivered so far (Chung et al. 2014; Wicker-Thomas et al. 2015). Cytosolic FASs appear to mainly catalyze the synthesis of saturated LCFAs, whereas microsomal FASs have been hypothesized to be more specific for mb-LCFAs (Juarez et al. 1992; Gu et al. 1997, see Fig. 2). Three FASs have been identified in *D. melanogaster*, two of which are expressed in the oenocytes (*FASN2* and *FASN3*) and one in the fat body (*FASN1*) (Chung et al. 2014). Knockdown of *FASN2* results in a strong decrease of mb-CHCs as well as an increase in *n*-alkenes without affecting desiccation resistance or overall CHC amounts (Chung et al. 2014; Wicker-Thomas et al. 2015). The authors thus speculate that *FASN2* is indeed microsomal and specifically catalyzes the production of mb-CHCs with an additional, less well-understood effect on *n*-alkenes (see Table 1). Furthermore, Wicker-Thomas et al. (2015) showed that although *FASN1* is expressed in the fat body, its knockdown still reduces the overall amounts of CHCs in *D. melanogaster*. *FASN1* therefore probably contributes to the overall pool of LCFA used for biosynthesis of CHCs (see Fig. 2). Knockdown of *FASN3* in wild-type *D. melanogaster* did not change the CHC profile but reduced desiccation resistance (Chung et al. 2014; Wicker-Thomas et al. 2015). The authors therefore suggest that *FASN3* catalyzes the production of other cuticular compounds besides CHCs that potentially contribute to waterproofing.

The phylogenies of FASs in multiple insect species suggest that the *FASN1/FASN2* genes of *D. melanogaster* originate from a gene duplication in the *Drosophila* genus (Finck et al. 2016; Moriconi et al. 2019). In case of such a duplication event, it is difficult to infer what role the common ancestor of both genes played in CHC production. *FASN1* and *FASN2* are expressed in different tissues and only *FASN2* has been shown to be involved in the production of mb-CHCs. The phylogeny of the FAS gene family across multiple insect orders also shows several lineage-specific expansions and Finck et al. (2016) suggest that the ability to synthesize mb-CHCs might have evolved independently. Orthologs of all three *D. melanogaster* FASs have been identified in the kissing bug *Rhodnius prolixus* (Hemiptera: Triatominae) with similar expression patterns (Moriconi et al. 2019). The ortholog to *FASN1* is predominantly expressed in the fat body, orthologs of *FASN2* and *FASN3* in the integument tissue. Knockdown of the ortholog to *FASN3* in *R. prolixus* resulted in an abnormal cuticle and reduced desiccation resistance (Moriconi et al. 2019). Furthermore, it led to reduced amounts of straight chain and mb-LCFA in the integument as well as a significant decrease in mb-CHCs while simultaneously increasing the amount of *n*-alkanes (Moriconi et al. 2019, Table 1). Similarly, knockdown of two FAS genes in the migratory locust *Locusta migratoria* decreased a subset of the mb-CHCs detected in this species, while both in- and decreasing the amounts of different *n*-alkane compounds with no apparent pattern (Yang et al. 2020). Two further putative FAS genes have been identified in the genome of the seaweed fly *Coelopa frigida*, though their direct impact on CHC variation has not been investigated as of yet (Berdan et al. 2019). Furthermore, seven FAS genes were identified in the German cockroach *Blatella germanica*, whose respective knockdowns revealed a significant effect of five of them on the CHC profile. Interestingly, knockdown of four *B. germanica* FAS genes actually increased the total amount of CHCs, whereas only one of them, *BgFas1*, substantially decreased both *n*-alkanes and mb-CHCs (Pei et al. 2019, Table 1). The above-mentioned examples indicate the challenges of transferring knowledge between taxa within the complicated phylogeny of the FAS gene family that has apparently undergone multiple duplications and neofunctionalizations (Finck et al. 2016; Moriconi et al. 2019). Their diverse functionalities are thereby far from restricted to CHC biosynthesis alone, but also include vital roles in lipogenesis, control of molting, and diapause induction (Tan et al. 2017; Lammers et al. 2019; Moriconi et al. 2019). This renders specifically characterizing and targeting FAS genes with a direct impact on CHC biosynthesis and diversification particularly difficult. Nevertheless, future investigations should put greater emphasis on identifying the specific properties of FAS genes that unambiguously associate them with CHC biosynthesis to contrast them more efficiently against other members of the FAS gene family.

### Desaturase (Desat)

The very first advances toward functional characterizations of CHC biosynthesis genes have been made with desaturase genes, governing the introduction of double bonds into hydrocarbon chains as basis for unsaturated CHCs (Cook 1985, see Fig. 2). These genes were primary targets due to both abundance and significance for sexual signaling of unsaturated compounds in *Drosophila* CHC profiles (Jallon 1984; Coyne and Oyama 1995; Marcillac and Ferveur 2004, see above). For instance, two desaturase genes with different substrate specificities, *desat1* and *desat2*, have been found to be responsible for introducing the first double bond into alkadienes and affecting alkadiene polymorphisms in different *D. melanogaster* strains (Wicker-Thomas et al. 1997; Coyne et al. 1999; Dallerac et al. 2000). Furthermore, a female-specific desaturase (*desatF*) has been revealed to be mainly responsible for affecting ratio and production of the female sex pheromone components 7,11- and 5,9-heptacosadiene in *D. melanogaster* by introducing a second double bond (Chertemps et al. 2006; Legendre et al. 2008). Concordantly, knockdown of *desatF* resulted in an increase of *n*-alkenes and a decrease of alkadienes in females (Chertemps et al. 2006, Table 1), demonstrating the strong correlation in the biosynthesis of these two unsaturated compound classes.

Helmkampf et al. (2015) investigated desaturases across the genomes of 15 insect species and found remarkable variation in this gene family, ranging from five paralogs in the bumble bee *Bombus terrestris* to 20 in the silk moth *Bombyx mori*. They also identified 16 orthologous desaturases in the genome of the jewel wasp *N. vitripennis*. Interestingly, Niehuis et al. (2011) found that three of the desaturases in the *N. vitripennis* genome co-localize with gene loci associated with variation of *n*-alkenes between *N. vitripennis* and its close relative *N. giraulti*. More recently, Berdan et al. (2019) uncovered two putative desaturases in the genome of the seaweed fly *Coelopa frigida*. Genetic variation in these genes aligned with variations in *n*-alkane to *n*-alkene proportions between populations, providing further evidence for the postulated desaturase-dependent link of *n*-alkane and *n*-alkene biosynthesis (Fig. 2). Future comparative studies should place larger emphasis on correlating desaturase gene counts and expression levels with occurrence and proportions of unsaturated CHC compounds in different insect taxa. This could help drawing direct conclusions and gaining a more general understanding on the expansions and reductions in this gene family and the corresponding impact on unsaturated CHCs.

### Elongase (ELO) enzyme complex

Elongases generally catalyze the first step in the elongation cycle of saturated and unsaturated LCFAs to VLCFs with chain lengths beyond 20 C (Blomquist and Bagnères 2010). The first elongase gene shown to have a function in CHC biosynthesis was the *D. melanogaster* female-specifically expressed *eloF* (Chertemps et al. 2007). Knockdown studies revealed a strong chain-length specificity of this gene, with upregulation of shorter-chain (C23-C25) *n*-alkanes, *n*-alkenes, and alkadienes and downregulation of longer-chain (C27-C29) CHC compounds (see Table 1). In contrast, three more recently identified putative ELOs in *D. melanogaster (CG30008, CG18609, CG9458)*, also expressed in males, showed no chain-length specificity. However, knockdown of these elongases affected abundances of several CHCs with no particular compound class specificities (Dembeck et al. 2015, Table 1).

Finck et al. (2016) suggest that in the common ancestor of insects the ELO gene family underwent a rapid expansion resulting in eleven ELO paralogs including eight that are insect-specific. After this ancient expansion, only very few additional duplication events could be inferred from the reconstructed ELO phylogeny (Finck et al. 2016). Three of the four ELOs of *D. melanogaster* that are associated with CHC profile variation can be found in a single clade, hinting at a recent expansion. Comparably long branches within this expansion indicate that these particular gene sequences show an increased divergence from homologous ELOs (Finck et al. 2016).

ELOs have been identified in many other insects as well, but the specific functions of the majority of them could not be assessed so far (Finck et al. 2016; Li et al. 2019a). For a fraction of them, however, a role in CHC biosynthesis could be assigned. For instance, a total of 20 ELOs have been identified in the brown planthopper *Nilaparvata lugens* (Li et al. 2019a). Nine of them have been found to be essential for survival, whereas the targeted knockdown of four of them most commonly resulted in decreased amounts of *n*-alkanes (Table 1). Interestingly, these ELOs have not been found to be closely related to the clade of ELOs that underwent expansion in *D. melanogaster* (Li et al. 2019a). Genetic variation in five other putative ELOs in the seaweed fly *C. frigida* corresponded with population-specific geographic variation in CHC chain lengths (Berdan et al. 2019). This matches well with predictions made by Chung and Carroll (2015), suggesting that ELOs are promising key candidates for causing CHC variation within and between populations through governing different compound chain lengths.

Elongation cycles are completed by a set of enzymes that can interact with each other to form elongase complexes, ultimately converting LCF acyl-CoAs through several intermediates into VLCF acyl-CoAs (Fig. 2). One of these enzymes, 3-keto-acyl-CoA-reductase, and its corresponding gene *spidey* have been found to be essential in oenocyte development in *D. melanogaster* (Parvy et al. 2012; Cinnamon et al. 2016). Targeted, oenocyte-specific knockdown of this gene resulted in almost completely eliminated CHC profiles indicating that it is essential for production of virtually all CHC compounds (Wicker-Thomas et al. 2015, Table 1). In the same study, two putative genes coding for 3-hydroxy-acyl-CoA-dehydratases are described (*Hacd1* and *Hacd2*) whose oenocyte-specific knockdowns resulted in moderate to strong decreases of several CHCs from all compound classes (Wicker-Thomas et al. 2015, Table 1). Lastly, another enzyme, trans-enoyl-CoA-reductase, and its corresponding gene, *Sc2*, were verified to be involved in the CHC elongation cycle as well. Targeted knockdown of Sc2 resulted in a significant decrease of most, but not all, CHC compounds in *D. melanogaster*, with no specificity pattern recognizable for any particular compound classes (Wicker-Thomas et al. 2015, Table 1). Unsurprisingly, gene expressions of *Spidey*, *Hacd1,* and *Sc2* have all been found to be upregulated in oenocytes (Huang et al. 2019). These findings indicate the substantial involvement of these genes as vital parts of the elongase enzyme complex in the biosynthesis of all CHC compound classes. However, functional characterizations in other insect taxa will be indispensable to determine the universality of their respective contributions to the elongation process.

### Fatty acyl-CoA reductase (FAR)

The whole FAR gene family has recently been studied in the genus *Drosophila* (Finet et al. 2019). The authors chose a phylogenetic approach paired with in situ hybridizations to identify FARs involved in CHC biosynthesis. They generally hypothesize that genes underlying CHC biosynthesis are rapidly evolving between closely related species, since CHC profiles have been shown to possess the capability to evolve rapidly as well (e.g., Jallon and David 1987; Chung and Carroll 2015; Rajpurohit et al. 2017). The number of FAR genes in 12 *Drosophila* genomes varied from 14 to 21, indeed indicating rapid evolutionary turnover with recurring gene loss and duplication events. The study found three of the four FARs expressed in adult oenocytes to be evolutionary unstable, which is also in line with their hypothesis as it hints at their potential to change more rapidly. Further, four out of five FARs were not crucial for viability. The authors therefore generally suggest that gains and losses of FARs could, by extension, allow for rapid diversification of CHC compositions in insects (Finet et al. 2019).

Moreover, two FARs identified by a genome-wide association study in *D. melanogaster* (*CG13091, CG10097*) have been shown to be directly associated with intraspecific CHC variation (Dembeck et al. 2015). Targeted knockdown of these genes increased the production of longer-chain CHCs in general, whereas shorter-chain *n*-alkene and mb-alkane quantities decreased (see Table 1). Curiously, this chain-length-specific up- and downregulation pattern is reminiscent of *eloF*, which functions more upstream in the CHC biosynthesis pathway (see above). Just as target specificity for different chain lengths of LCF acyl-CoAs has been demonstrated for *eloF* (Chertemps et al. 2007), the regulation pattern of these two FARs hints at a similar target specificity for VLCF acyl-CoAs (see Fig. 2).

Two other FAR genes have been functionally characterized in *D. melanogaster*, *FarO*, and *wat* (*waterproof*). *FarO* is specifically expressed in larval oenocytes and regulates oenocyte growth, with no demonstrated effect on CHCs in adults (Cinnamon et al. 2016), whereas *wat* has been found to be essential for tracheal gas filling (Jaspers et al. 2014). Thus, both have no apparent association with CHC production, variation, or composition (Cinnamon et al. 2016). Interestingly, however, a homolog to *wat* in the brown planthopper *Nilaparvata lugens*, *NlFAR7*, is responsible for water-repellent properties of the cuticle and its knockdown resulted in a reduction of overall CHC quantities by almost half (Li et al. 2019b, see Table 1). Knockdown of another FAR gene identified in *N. lugens*, *NlFAR9*, also reduced CHC levels in adult planthoppers, albeit apparently only affecting longer-chain *n*-alkanes (above C 26), providing further evidence that chain-length-specificity potentially constitutes a common trait in FARs (Li et al. 2020).

Of the further 15 FARs identified in *N. lugens*, ten were found to be essential for cuticle shedding and wing expansion during molting with a lethal knockdown phenotype, while others had a marked detrimental effect on female fertility (Li et al. 2020). This illustrates a much farther-reaching and diverse functionality of insect FARs than previously appreciated. Perhaps most interestingly, knockdown of *NlFAR7* also slightly increased gene expression of ACC, whereas *NlFAR9* knockdown slightly increased the expression of a FAS gene in *N. lugens* (Li et al. 2020). This revealed previously undiscovered correlations of the expression patterns of CHC biosynthesis genes and warrants further investigation of the underlying gene expression networks.

### Cytochrome P450 decarbonylase (CYP4G)

It has long been assumed that FARs directly reduce VLC acyl-CoAs to VLC aldehydes (Reed et al. 1995). However, this view has recently come into question. Namely, it has been demonstrated that P450 decarbonylases of the enzyme subfamily CYP4G, thought to only catalyze the oxidative decarbonylation of VLC aldehydes to CHCs in the last biosynthesis step (Feyereisen 2012; Qiu et al. 2012), can also directly reduce alcohols to aldehydes (MacLean et al. 2018). In addition, all insect FARs biochemically characterized so far have only been experimentally validated to be capable of converting acyl-CoAs into primary alcohols, and not further to aldehydes (Cinnamon et al. 2016; Hu et al. 2018). This, in turn, renders the extended functionality of CYP4G P450 decarbonylases for both the oxidization of alcohols to aldehydes and then the oxidative decarbonylation of the latter to CHCs very likely (see Fig. 2). Due to this dual role, the CYP4G enzyme subfamily constitutes a particularly vital component of the CHC biosynthesis pathway. Fittingly, at least one *Cyp4g* gene could be identified in all insect genomes screened to date (Qiu et al. 2012; Feyereisen 2020). Moreover, the general function of converting aldehydes (or alcohols) to hydrocarbons has already been demonstrated in several insect CYP4G enzymes: Cyp4g2, house fly *Musca domestica* (Qiu et al. 2012); Cyp4g16, mosquito *Anopheles gambiae* (Balabanidou et al. 2016); Cyp4g11, honey bee *Apis mellifera* (Calla et al. 2018); Cyp4g55 and Cyp4g56, mountain pine beetle *Dendroctonus ponderosae* (MacLean et al. 2018). Even more direct evidence has been delivered for a couple of *Cyp4g* genes whose targeted knockdown resulted in a substantial decrease in total CHC amounts (see Table 1): *Cyp4g51*, pea aphid *Acyrthosiphon pisum* (Chen et al. 2016); *LmCYP4G102*, migratory locust *Locusta migratoria* (Yu et al. 2016) and *Cyp4g1* in *D. melanogaster*, which additionally lead to high desiccation susceptibility and reduced viability (Qiu et al. 2012). It has been suggested that CYP4Gs are nonspecific regarding the chain length of their substrate, contrasting the chain-length specificities hinted at for FARs and *eloF* (see above). This would also suggest that CYP4Gs constitute highly conserved, evolutionary stable elements in the CHC biosynthesis pathway, essential for catalyzing its last steps. However, clearly more direct experimental validation is required to demonstrate the importance and universality of CYP4Gs in CHC biosynthesis.

## Future directions for unraveling CHC biosynthesis genes

### Novel candidate genes with potential impact on CHC biosynthesis

Recently, a surprising correlation between *Cyp4g* gene expression and oenocyte-specific expression of a receptor gene for the insect oxytocin/vasopressin ortholog inotocin was found in the carpenter ant *Camponotus fellah* (Koto et al. 2019). This study provided indirect evidence for the involvement of the inotocin signaling pathway in regulating CHC quantities. Furthermore, three other *cyp* genes apparently associated with CHC variation were found in *D. melanogaster* that are not direct members of the specific *Cyp4g* gene subfamily (Dembeck et al. 2015). Instead, those *cyp* genes all belong to other clades of the much larger and extremely diverse Cytochrome P450 gene family, of which none had hitherto been associated with CHC biosynthesis or variation (Chung et al. 2009; Feyereisen 2012). Their function apparently differs from the *Cyp4g* gene (see above), as their knockdown not only resulted in down-, but also upregulation of CHC compounds. These changes were sex-specific to some extent, but not for any particular CHC compound class (Table S1). Further genes identified for their impact on CHC variation in *D. melanogaster* include four coding for peroxidases, one for a transmembrane protein affecting desiccation resistance (Desi) and one for a palmitoylation enzyme (App) that can modify cytoplasmic proteins by adding palmitic acid residues (Dembeck et al. 2015, see Table S1). In another recent study on *D. melanogaster*, two genes generally associated with melanin biosynthesis, *ebony* and *tan*, have also been associated with CHC variation (Massey et al. 2019). Specifically, CHC profiles of *ebony* loss-of-function mutants were biased toward more long-chained CHCs beyond 25 C, whereas CHC profiles of *tan* loss-of-function mutants had more short-chained CHCs below 25 C. Whether these CHC profile alterations are only secondary consequences of other *ebony*- and *tan*-dependent phenotypic effects (e.g., changes in the cuticle’s pigmentation) remains unclear. Intriguingly however, the observed changes in CHC compositions are again reminiscent of the chain-lengthspecific effects hinted at for FARs and *eloF* mentioned above.

The biochemical mechanisms by which all of these genes potentially regulate or contribute to CHC biosynthesis are completely unknown so far and can only be speculated upon. These examples illustrate how fruitful it might prove to be to pursue avenues not yet taken in the search for potential CHC biosynthesis candidate genes. This is especially relevant considering that certain details in the CHC biosynthesis pathway still remain largely unknown (see Fig. 2). For instance, the transport mechanisms for crucial precursor molecules into the onoecytes, e.g., LCFAs from the fat body or amino acids for 2-methyl-branched CHC synthesis, have not yet been resolved (Blomquist and Bagnères 2010; Wicker-Thomas et al. 2015). Also, the exact mechanisms giving rise to the myriad of different mb-CHCs occurring across most analyzed insect taxa are far from fully understood. Related to this, two genes with postulated Lipoamide acyltransferase activity, *CG5599* and *CG8680*, had an upregulatory effect on *Drosophila* CHC production (Dembeck et al. 2015, see Fig. 2 and Table 1). Since the enzymatic activity of lipoamide acyltransferases can result in the integration of methyl branches into lipoamides (Chuang et al. 1984), this might constitute an additional mechanism of integrating methyl-branches into CHCs. Furthermore, the conversion from LCFAs to LCF acyl-CoAs has not been completely elucidated, with an exact molecular characterization of a postulated enzyme with LCFA CoA-ligase/-synthetase activity still lacking. Most prominently, though, it remains unclear as to how specific the production of different CHC compound classes can be regulated. The vast majority of genes identified so far with a functionally demonstrated impact on CHC variation simultaneously affect multiple CHC compound classes at once (compare Table 1 and Fig. 2). This renders the disentangling of this delicate biosynthetic network with various apparently linked genetic regulators very challenging, particularly if the focus lies on individual CHC compound classes.

### Sex-specificity and gene expression levels

Evolutionary genetic studies on CHC biosynthesis also need to account for sex-specific differences in the regulation of CHC biosynthesis (e.g., Foley et al. 2007; Dembeck et al. 2015; Luo et al. 2019). Despite sharing the same genes in principle, sexes can show pronounced differences in their CHC profiles, qualitatively as well as quantitatively (e.g., Buellesbach et al. 2013; Berdan et al. 2019; Berson et al. 2019a). As has already been demonstrated, numerous CHC biosynthesis genes seem to show a strong expression bias in one particular sex with a pronounced sex-specific impact on the respective CHC profile (Chertemps et al. 2006, 2007; Dembeck et al. 2015, see Table 1 and S1).

Generally, when comparing taxa with mostly quantitative CHC differences, assessing gene expression level differences and correlating them with CHC profile variation might ultimately prove to be a more powerful approach than targeted knockdown studies. In addition, in situ hybridization targeting expression of genes in oenocytes and other potentially CHC-related tissues (e.g., the fat body) has the potential to greatly help complementing insights from correlative gene expression data, as has already been successfully demonstrated in a few case studies (e.g., Finet et al. 2019; Koto et al. 2019).

Together this raises the question whether most differences in CHC profiles can be explained through differential regulation of the underlying genetic networks rather than presence or absence of biosynthetic genes. It becomes increasingly clear that fine-tuning gene expression, e.g., through transcription factors, post-translational as well as epigenetic modifications, very likely plays a fundamental role in generating CHC variation. To the best of our knowledge, however, the influence of particularly epigenetic factors on CHC profiles has not been investigated at all so far. Furthermore, quantitative genetic studies hint at additive effects of many genes with the potential to cumulatively impact CHC variation, complementing studies on heritability and genetic correlations between individual CHC compounds (Foley and Telonis-Scott 2011; Berson et al. 2019b; Walsh et al. 2020). This would limit the significance of the candidate gene approach for resolving the genetic basis of CHC biosynthesis. This also hints at a much more complex, multifaceted and intricately interwoven genetic network with various small and large effects on CHC variation than previously appreciated.

### Phenotypic plasticity and nongenetic factors

Another confounding factor in studying CHC genetics is the apparently large degree of phenotypic plasticity characterizing CHC profiles (Otte et al. 2018). Nongenetic factors contributing to CHC diversity could potentially overshadow the detectable impact of individual biosynthesis genes, hampering attempts to fully resolve the explicit contributions of each individual gene. Several studies have revealed a remarkable adaptability of CHC profiles to diverse biotic and abiotic conditions in different insect taxa (e.g., Rajpurohit et al. 2017; Buellesbach et al. 2018b; Menzel et al. 2018). Also, since CHC biosynthesis is linked to the uptake of amino acids (Dillwith et al. 1982; Chase et al. 1990) and has been shown to be influenced by several metabolic pathways, such as the citric acid cycle and fatty acid synthesis (Barber et al. 2005; Blomquist and Bagnères 2010), diet can contribute to variations in CHC profiles as well (e.g., Fedina et al. 2012).

Finally, changes in the microbiome can alter CHC profiles via different mechanisms, such as direct degradation of CHCs (Napolitano and Juárez 1997), or the production of precursor metabolites (Guo et al. 1991). Deciphering the actual conserved genetic signatures in CHC profiles not caused by environmental, dietary, or microbial factors is thus a very challenging but indispensable task for gaining a more holistic perspective of CHC biosynthesis.

### Taking a wider phylogenetic approach

As evident from this review, most of the genetic investigations undertaken so far have remained very specific to the *Drosophila* genus and are not necessarily representative of CHC biosynthesis across wider phylogenetic boundaries. For instance, in other insect taxa, particularly Hymenoptera, mb-CHCs tend to occur in far higher and more dominant quantities than in *Drosophila* (e.g., Buellesbach et al. 2013; Kather and Martin 2015; Sprenger and Menzel 2020). Therefore, this compound class has so far largely been neglected in favor of the more *Drosophila*-specific *n*-alkenes and alkadienes (Jallon and David 1987; Dallerac et al. 2000; Ferveur 2005). This is unfortunate due to the particularly high potential for encoding chemical information in mb-CHCs (Chung and Carroll 2015) and several studies that have already demonstrated their significance in chemical communication (e.g., Holman et al. 2010; Spikes et al. 2010). However, the overreliance and bias toward *Drosophila* have contributed to the comparably limited understanding about the genetic factors governing variation in this particular compound class (Blomquist and Bagnères 2010; Niehuis et al. 2011; Dembeck et al. 2015). More recently, several genetic studies on other insects, most prominently in the orders Orthoptera and Blattodea (see Table S1), have contributed to broaden our understanding of CHC genetics beyond *Drosophila*. It will be very helpful to greatly expand on these findings in future studies, incorporate more insect taxa and implement more comparative approaches to shed light on the conserved blueprint of CHC biosynthesis on a larger phylogenetic scale.

Through an even wider evolutionary lens, the cuticles of insects and plants share remarkable structural similarities in their hydrocarbon compositions, even down to generally similar biosynthetic pathways (Kunst and Samuels 2003; Bernard and Joubès 2013). Moreover, the ability to synthesize hydrocarbons through comparable pathways despite partially very different enzymatic complexes and reactions has also been demonstrated in several microorganisms and fungi (Ladygina et al. 2006; Schirmer et al. 2010). Intriguingly, hydrocarbons in these vastly different organisms are apparently capable of serving similar protective functions against varying environmental conditions (e.g., Weete 1972; Bagaeva and Zinurova 2004). However, integrative studies have only just begun to draw comparisons between potentially convergent factors regulating biosynthesis and variation of structurally similar hydrocarbon compounds across such vastly divergent domains of life (e.g., Marsh and Waugh 2013; Barbero 2016). It will be particularly interesting to see if these similarities partially originate from conserved genetic elements recruited for comparable biosynthetic pathways that have led to such striking convergent phenotypic similarities. We are clearly only at the beginning of unraveling this remarkable phenomenon and understand its evolutionary and ecological implications.

## Conclusions

We have come a long way from uncovering the first genetic factors underlying sex- and species-specific CHC variation to now being able to elucidate the contributions of individual genes to minute variations in CHC compositions. However, we are still far from fully and conclusively understanding the extent of the genetic framework underlying CHC biosynthesis. Specifically, apart from a few well documented case studies of unsaturated CHCs in *Drosophila*, we have not yet resolved individual genetic factors exclusively governing the synthesis of particular CHC compound classes, let alone single compounds. This is partially due to the continuing lack of insight into differences between conserved versus more taxon-specific genes regulating CHC biosynthesis, in turn preventing us from developing a more general blueprint of this fundamental biological process. Therefore, the most promising avenues of research lie in incorporating an increasing number of insect taxa with a larger focus on integrating comparative genome-wide, phylogenetic and functional genetic studies. Ultimately, this will not only help us to unravel the more general aspects of CHC genetics but also potentially answer interesting evolutionary questions about the origins, diversifications, and expansions of these essential phenotypic traits.

## Supplementary information

Table S1
